# Privacy-Related Context Information for Ubiquitous Health

**DOI:** 10.2196/mhealth.3123

**Published:** 2014-03-11

**Authors:** Antto Seppälä, Pirkko Nykänen, Pekka Ruotsalainen

**Affiliations:** ^1^Center for Information and SystemsSchool of Information SciencesUniversity of TampereTampereFinland

**Keywords:** ubiquitous health, privacy, context information, trust, policy

## Abstract

**Background:**

Ubiquitous health has been defined as a dynamic network of interconnected systems. A system is composed of one or more information systems, their stakeholders, and the environment. These systems offer health services to individuals and thus implement ubiquitous computing. Privacy is the key challenge for ubiquitous health because of autonomous processing, rich contextual metadata, lack of predefined trust among participants, and the business objectives. Additionally, regulations and policies of stakeholders may be unknown to the individual. Context-sensitive privacy policies are needed to regulate information processing.

**Objective:**

Our goal was to analyze privacy-related context information and to define the corresponding components and their properties that support privacy management in ubiquitous health. These properties should describe the privacy issues of information processing. With components and their properties, individuals can define context-aware privacy policies and set their privacy preferences that can change in different information-processing situations.

**Methods:**

Scenarios and user stories are used to analyze typical activities in ubiquitous health to identify main actors, goals, tasks, and stakeholders. Context arises from an activity and, therefore, we can determine different situations, services, and systems to identify properties for privacy-related context information in information-processing situations.

**Results:**

Privacy-related context information components are situation, environment, individual, information technology system, service, and stakeholder. Combining our analyses and previously identified characteristics of ubiquitous health, more detailed properties for the components are defined. Properties define explicitly what context information for different components is needed to create context-aware privacy policies that can control, limit, and constrain information processing. With properties, we can define, for example, how data can be processed or how components are regulated or in what kind of environment data can be processed.

**Conclusions:**

This study added to the vision of ubiquitous health by analyzing information processing from the viewpoint of an individual’s privacy. We learned that health and wellness-related activities may happen in several environments and situations with multiple stakeholders, services, and systems. We have provided new knowledge regarding privacy-related context information and corresponding components by analyzing typical activities in ubiquitous health. With the identified components and their properties, individuals can define their personal preferences on information processing based on situational information, and privacy services can capture privacy-related context of the information-processing situation.

##  Introduction

### Overview

Ubiquitous computing makes it possible to collect all kinds of data anywhere and anytime [1] and allows integration of health care delivery and services into people’s everyday lives [2,3]. This paper builds on a conceptual framework [4] in which ubiquitous health is defined as an open and dynamic ubiquitous information space. The space is presented as digital systems that consist of one or more information systems, their stakeholders, and environments. These systems create a dynamic network that offers and provides services to citizens. In the information space, individuals and service providers can select, tailor, and combine services and systems that belong to the network. To enable access to personal information, individuals and providers need to discuss trust, privacy level, and proffered service.

Ubiquitous health services can be offered by providers that are licensed and regulated by medical ethical codes and health care–specific legislation and other juridical norms and by actors that are not affected by health care–related regulations. To separate these two groups, we divided them as regulated health care services and other services. Providers offering regulated health care services have strict defined responsibilities and obligations concerning service provision, care, professionals, documentation, and information processing. There are also general regulations on privacy and security requirements (eg, data protection and processing directives) and business domain–specific regulations. Regulations cover laws; norms; good practice guidelines; and other rules controlling, constraining, or limiting activity of participants. These regulations can affect ubiquitous health services but they often do not meet the challenges of technological innovations well.

In ubiquitous health, trustworthiness and privacy are key challenges [4-6]. There are privacy threats created by autonomous and hidden processing of information and rich contextual metadata. There is no predefined trust between participants, and the business objectives, needs, interests, and policies of stakeholders may be unknown to the individual [4]. Information in ubiquitous health is highly sensitive and confidential, and the existence of services and actors that are not strictly regulated by health care-specific legislation creates threats and risks for individual privacy. In addition, information processing can happen in multiple systems and situations with different regulations, and risks of secondary use exist. The lack of predefined trust and privacy risks emphasizes the importance of an individual's ability to control his or her privacy.

For trusted information processing in ubiquitous health, we follow the principles presented in Ruotsalainen et al [4] and according to them, an individual should have the right to verify dynamically the trustworthiness of the ubiquitous health network and any system that requires or processes the individual’s personal information for secondary purposes; control personal health information processing, inside systems and between them; be notified of all situations and contexts in which personal information is collected, processed, stored, and/or disclosed; and create situation-specific, context-aware, and granular personal privacy and trust policies, which control how personal information is collected, processed, disclosed, shared, stored, or destroyed.

Systems and stakeholders should have the responsibility to ensure trust verification by publishing their privacy policies and environmental and contextual features; openness of interests, business needs, and policies as well as their relationships with other systems; and transparency of information processing.

To protect his or her rights, an individual needs information about privacy, that is, privacy attributes, to define his or her personal privacy preferences. Privacy attributes enable privacy to be a concrete issue for individuals. In Nykänen et al [7], we defined privacy attributes as benefit, benevolence, capability, competence, confidence, context, reliability, and value. Privacy attributes and their contents have not generally been researched widely. The focus in this study is the context attribute, which refers to the situation in which data are created or processed. The objective is to analyze and define privacy-related context information components and their corresponding properties.

**Figure 1:**
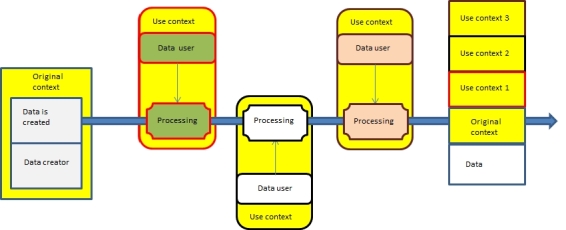
Data continuum and context information.

When data are created, a continuum of data is born. During the different processing situations, data or its properties may change. Original context refers to a situation when data are created. In various use contexts and processing situations, context information is incrementally created and it describes the current context and enables tracking of the context history. Thus, data have embedded context information that can be used by privacy services for trust calculation and to decide whether processing is allowed (Figure 1).

An individual’s privacy preferences can be implemented with adaptable privacy policies. In previous work [8], we concluded a formula for privacy policies to contain (1) trust information that is a value of a system- or environment-specific calculation of regulatory compliance and trustworthiness; (2) sensitivity of the data; (3) situation of the information use; and (4) purpose of the data collection or use.

Policy formulation is a decision process in which an individual selects privacy rules and services and how much information can be traded compared to the offered service and the level of privacy attributes. In this study, our hypothesis is that context information enables formulation of context-aware privacy policies hence enabling trustworthy processing of personal health and wellness information and realizing individuals’ rights for privacy in ubiquitous health. In Ruotsalainen et al [8], we presented a privacy architecture that could use context information in trust calculation and in context-aware privacy policies to control an individual's personal information. With this study, we add knowledge to our earlier research by studying the privacy-related context information and by defining the corresponding components and their properties that support privacy management in ubiquitous health.

### Prior Work

#### Privacy and Trust

Privacy refers to an individual’s ability to control information about him- or herself [9]. Privacy is a very personal concept and dependent on the context, because it may vary among jurisdictions, cultures, economies, time, and individuals [10-12]. Smith et al [13] claim that privacy is so bound to the specific context that it cannot be conceptualized as a single and unambiguous concept; rather it should be treated as a set of interests. Clarke [14] argues that it is useful to understand privacy as the interest of keeping personal space free from inference and has divided privacy into four dimensions: person, personal behavior, personal communications, and personal data. Information privacy means that personal information should not generally be available to other persons or organizations and an individual should have major control or influence over the personal data controlled by others and its use [14]. In this research, we refer to privacy as an individual’s personal view within the legislative boundaries.

Trust is a concept closely related to privacy, and usually, the higher the value of trust, the lower the need for privacy [4]. Trust implicates the willingness to share personal information with others [15]. Schoorman et al [16] emphasize that trust is based on a relationship and the level of trust expresses the level of risk an individual is willing to take. Abdul-Rahman and Hailes [17] have defined three characteristics of trust: (1) trust is subjective, (2) actions we cannot monitor affect trust, and (3) trust level is dependent on how others’ actions affect our actions. Several trust models has been developed for calculating trustworthiness [16,18-20].

Ubiquitous computing systems should be open and dynamic, because pre-identification of participants is impossible and they might change regularly [21]. In these kinds of distributed environments, collaboration is vital because multiple systems together try to achieve goals and perform tasks and it is crucial for systems to know which entities they should or should not interact with [22]. Traditional privacy and security solutions are not adequate for ubiquitous environments because there is no central control or predefined users or policies [19,21,23]. Privacy and security architecture and decisions need to be based on trust and its properties [19,21,24].

#### Context and Context Awareness

Context has been mostly defined with user profile, user emotion, and user location and identities of nearby people and objects and changes to those objects [25-28]. According to Dey et al [29], the three most relevant entities are places, people, and things. These entities have to be considered from different viewpoints such as location, activity, and identity. Dourish [30] proposes that context and content cannot be separated; the context arises from the activity itself and it cannot be an external description of the setting. He claims that context is a relational, interactional property between objects and activities and the scope of features must be defined dynamically [30]. Dey and Abowd ([28], pp. 3-4) defined context as: “Context is any information that can be used to characterize the situation of an entity. An entity is an individual, place, or object that is considered relevant to the interaction between a user and an application, including the user and applications themselves.”

This definition is open and it considers that any information that is relevant for information processing in a situation can be used as a context. Context information can, for example, be information about the user, device, environment, or situation. Thus, it is meaningful to talk about context related to something that exists. There are three main uses for context information [29]: (1) presenting information and services to a user or using context to propose actions to be performed, (2) execution of a service automatically on behalf of the user, and (3) applications can tag context to information for later retrieval.

In context-aware computing, applications and systems are able to perceive their surroundings and environment, adapt according to the context, and perform autonomously. Context awareness refers to adaptability, which means that applications and systems exploit perceived context information and adapt their behavior accordingly [31]. In this view, context information is information that enables behavior modification based on this information and its relations. The systems, applications, and entities have to define the scope themselves.

According to Viswanathan et al [32], the key point for successful ubiquitous health is context awareness, and there are already several context-aware applications in the health and wellness domain. A lot of research has been done to support personalized actions and services in home care, chronic disease management, and ambient assisted living [33-37] with different personalized health status, body sensor networks, activity or behavior monitoring, decision support, and reminder applications [32,33,35-40]. In the hospital environment, many professionals are very agile, and context-aware technologies may help by personalizing services for them by location, time, and social context [41]. According to previous studies [33,42], there are several experiments on context-aware computing that have been created in hospital environments to improve patient record management, communication among professionals, and information sharing by including context awareness in patient room equipment.

#### Policies

In ubiquitous environments, privacy requirements can be expressed with policies. Privacy policy can be understood as a personal statement on privacy. With policies, individuals can set computational rules explicitly stating their personal privacy preferences on how their information can be processed, used, disclosed, and shared [21,43,44]. Policies are typically expressed with a policy language [45]. To enable personal privacy policies with computational rules requires definition of privacy attributes. Privacy policies can be implemented with setting values on privacy attributes. Context-aware policies based on context information enable dynamic adaptation of privacy control strategies and tailored privacy decision support services. A technique called sticky policy enables attaching policies into data to ensure that data are processed according to an individual’s wishes [44].

Behrooz and Devlic [46] propose a context-aware privacy policy language based on two design considerations: (1) situations and privacy rules are defined separately, and (2) a context requestor can be specified based on its identity or social relationship to a user. These principles mean that privacy policies are set for different situations. Ghosh et al [47] presented a semantically rich policy-based system that can reason on user’s context and thus protects a user’s privacy dynamically during runtime. Schaub et al [23] presented a privacy context model with three major entities—user, user’s environment, and user’s activities. Their model takes into account information, physical, and territorial aspects of privacy. Blount et al [48] proposed a context-dependent policy model in which field context contains information when conditions for the policy are valid. These values may be from either the subject or the requestor.

## Methods

### Scenarios and User Stories

Scenarios are means to describe the system’s intended usage. Scenario-based design techniques produce descriptions of how people do things and how they can accomplish different tasks with the system. With scenarios, designers can find new ways of doing things and new things to do. Scenarios capture goals, entities, behavioral information (eg, actions, activities, and events) and what people are trying to accomplish with the system [49,50]. They can also describe different related actors with their own objectives. Typically, scenarios have a plot that consists of several events, things that happen during activities, changes in the setting, etc. Scenarios are work-oriented analysis methods; thus, they are suitable for our purposes, because we are analyzing typical activities of an individual in an ubiquitous health environment to recognize the needs for context information.

In our previous articles, we analyzed privacy threats and the principles for trusted information processing [4], defined privacy attributes [7], and analyzed the requirements for information that should be used in privacy policy formulation and common threats and challenges concerning privacy in ubiquitous health [8]. Our previous results created the framework for the scenario development and analyses and for the requirements for context information. In this research, we created scenarios that were based on materials collected in our earlier empirical research on personal wellness [51-53] performed with focus groups and literature studies focusing on health and wellness activities and technical applications on chronic disease management, self-health management, ubiquitous health, and wellness approaches. Scenarios were designed to capture the characteristics of different situations, such as a general wellness management situation without any specific needs and a specific setting with a chronic disease. With scenarios, we could identify a wide selection of typical activities in ubiquitous health.

We first created two textual scenarios describing the main actors, their backgrounds, and current health and wellness situations and next, we determined the main goals, activities, and entities. Then, we further divided both scenarios into 10 user stories that described in more detail the activities and services the individuals needed in their situations. Each user story focuses on 1 activity of a scenario and it is a short textual and informal description of a user case. Because context arises from activity [30] with the user stories, we could capture activities in ubiquitous health to identify information-processing situations and privacy-related context information.

At first, scenarios described typical wellness approaches emphasizing services that are not regulated by health care regulations, for example, lifestyle management and health-related behaviors. The objective was to recognize activities and entities outside regulated health care services. Then we approached chronic disease management scenarios with a focus on identifying collaboration between regulated health care services and personal attempts to manage health outside the provider networks with other services. These scenarios were analyzed to recognize activities and information-processing situations. To summarize, these scenarios helped us to analyze the aspects of two different situations in ubiquitous health: (1) ubiquitous health without regulated health care providers’ participation; and (2) ubiquitous health with regulated health care, for example, service portfolio is a combination of services produced by a regulated health care provider(s) and other health and wellness providers.

### An Example Scenario

As an example, we present the following scenario. Peter is a 23-year-old healthy student who begins to feel tired and ill and he decides to seek help from student health services. After a few tests and doctor visits, Peter is diagnosed with type 2 diabetes mellitus. From now on, Peter has to pay attention to his habits and choices concerning healthy living for the first time in his life. We divided this scenario into more detailed user stories describing activities related to chronic diseases in a ubiquitous health environment. In Table 1, we present an example analysis of a user story. In Table 2, we present a detailed example of a single activity in ubiquitous health with its related privacy concerns, Peter’s policies, and the context information that a policy example requires.

**Table 1:** An example analysis of a user story in chronic disease scenario. User story 2.1: Peter receives a medical device with sensors to manage and care for his disease and automatically measure and monitor his condition. Devices can also automatically inform his doctor about the results and major changes.

Role	Individual and information controller with rights for privacy, to control processing and secondary use of information. Peter can decide who can access data created by the device. Peter needs privacy policies to control his own personal health system (PHS) use and the information it contains.
Activities	Data is created in the sensors and transferred to PHS. PHS analyzes the information and compares it to past information. PHS informs Peter’s doctor about a major change in a value. Doctor accesses the information and makes a medical decision.
Environment	Anywhere. No health care–specific regulations concerning the environment. Information sharing is based on Peter’s known consent and privacy policies. All information created by the certified device is trusted. The device is regulated by specific legislation (eg, the European Union directive on medical devices). In case of a major change in measurement information, regulated health care service will participate and then the environment will be strictly regulated by health care–specific regulations.
Information systems	Medical device, Peter’s own PHS and possibly electronic health record system. Sensor and measurement data is stored in PHS and Peter’s health records are in regulated electronic health record system. Peter has total control over his PHS.
Stakeholders	Peter, medical device, PHS, and licensed medical professional (doctor) with responsibilities concerning care and patients privacy
Services	Certified medical device measuring blood sugar levels PHS diabetic information analysis Regulated health care service activated by Peter’s PHS in case of a major change in Peter’s measurement values
Information content	Measurement and monitoring data from sensors and medical device Health and wellness information in PHS is controlled by Peter. The medical information is regulated in health care organization’s electronic health record system.
Original context of the information	Information is created by a certified medical device controlled by Peter. The environment does not have any specific domain regulations. Information is in Peter's control and he has full rights for it. Peter's personal context-aware privacy policies are the main source for limitations and constraints on information processing.
Requirements for context properties	Peter’s PHS is a trusted information system in his control so it has full processing rights and can activate other services if needed following Peter’s privacy policies. Peter has defined in his policies that different measurement and sensor data is very sensitive and sets limitation for what purpose information can be used. In other cases, PHS cannot grant access to information without Peter’s authorization. Other than regulated health care, services have to share their principles for information processing, security and privacy policies, and for what purpose they want to process the information.

**Table 2:** An example analysis of an activity: data is created in the sensors and transferred to the PHS.

Privacy challenges and threats [8]	Peter’s policies	Required context information for policy 1
Lack of awareness	1. Peter thinks that this kind of data is highly personal and can only be accessed automatically by a health care professional participating in Peter’s care service.	Situation: activity, processing type, actor, target, information sensitivity, and purpose for processing
It is difficult to know how data is used in the future	2. To use the data, transparency of processing is needed; therefore, the provider has to publish detailed privacy and security policies and allow third-party auditing.	Environment: general privacy and security regulations, location, and society
Relationships between systems may be unknown	3. To prevent secondary use, copying data is not allowed. If copying is required, Peter has to be notified and his known consent is required.	Service: type, role, provider, location, and objective
Potential secondary use of information	4. Health care professionals are not allowed to disclose data without Peter’s known consent.	Individual: role, rights to control information, relation to the activity, confidentiality requirements
Users want to control how systems use personal health information		Stakeholder: identity, type, role, purpose, and justification for processing
How to guarantee that data is processed following the legal constraints and according to the individual’s policies		IT system: identity, type, controller

## Results

In an open and dynamic ubiquitous health information space, there are no possibilities to predefine entities or activities and most aspects of information processing are dynamic. In the scenarios and user story analyses, we recognized how different activities are reasons for information-processing situations in ubiquitous health, how several entities can create and use information, and how the same information can be used later to support different activities. In addition, scenarios showed that activities could happen autonomously with information systems even without human participation; for instance, based on some measurement of vital signs or monitoring of data. Thus, information processing happens because some entity performs an activity in a certain environment. Situation describes this occurrence and therefore is chosen as the core component defining privacy-related context information. It is linked to a certain activity; that is, the reason for information processing. Context information needs to include the whole situation and all participants because of the dynamic nature and limitations in predefining activities and stakeholders in ubiquitous health.

As a result of our scenarios and user stories, we present the two kinds of basic models for ubiquitous health: ubiquitous health without regulated health care providers, and ubiquitous health with regulated health care service providers.

The first case is an open environment with multiple entities with different kinds of domain environments and interests. All participants are by definition untrusted. Health care–specific regulations do not apply, but regular privacy and security legislations set limitations for information processing. In addition, different domains may have their specific legislations (eg, social care, wellness services, medical devices, or pharmacy). Environment and entity-specific regulations and an individual’s personal context with privacy preferences are necessary for adaptable privacy policies. An individual’s role, environment, and privacy requirements may vary between used services or information systems and information sensitivity influences heavily on personal policies. An individual’s rights to control data and information must be discussed with service providers.

In the second case, there are also entities that are affected by health care–specific regulations. Depending on who or what provides service and/or controls information, there might be strict health care–specific regulations for service provision, organizations, professionals, information systems, and information processing. Regulated health care services are to some extent trusted and privacy threats and risks occur especially when information is transferred from them or processed beyond their authority. It is very critical to capture who is responsible for what, where and how services are provided, what information and sources are used, how sensitive the information is, and who controls participating information systems.

In a previous study [4], we defined ubiquitous health to be composed of services, information systems, stakeholders, and their environments. In addition to these, we have to capture the contexts of the information-processing situation and its object and/or subject. We should capture the following components and their properties on privacy, regulations, and requirements for trusted information processing: what happens (situation); who is the subject or the object (individual); what services are related to the situation (service); where this situation happens (environment); what social actors are active in the situation (stakeholder); and what computational entities participate (IT system).

In this research, the properties of the privacy-related context information components and their properties are derived by combining the results of the scenario analyses and the principles and requirements presented in the earlier research. We analyzed the results of the scenario analyses to explicate concrete properties for our components. In the example, we derived the context information that is needed to fulfil the requirements for policies and to minimize known privacy threats. In this example, policy 1 in the Table 2 means that Peter sets a general policy that data created by sensors is highly sensitive and can only be automatically accessed by health care professionals participating in his care. Peter has total control over his data and the future use of data is based solely on Peter’s wishes. The situation occurs when a regulated health care professional tries to access Peter’s data to support Peter’s care and to follow his condition. To manage his privacy, Peter needs information about the data user’s environment and processing wishes. If parties other than a health care professional in Finland taking part in Peter’s care service want to access the data, Peter’s known consent is required. The data user is a regulated health care professional in Finland; that is, predefined as somewhat trusted and he/she can use the information only to make medical decisions and to follow Peter’s condition. The data can only be accessed within Peter’s PHS and the data cannot be copied or distributed. From the example, we can see how Peter needs several kinds of context information to create the example policy.

**Figure 2:**
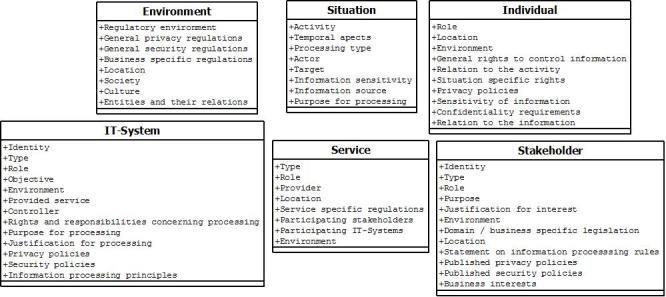
Privacy-related context information components and their properties for ubiquitous health.

From the scenario analyses, we have defined the properties that are needed to fulfil the principles of trusted information processing and requirements set for privacy formulation concerning context information (Figure 2).

A situation describes information processing that happens in a certain context because of some activity and by/for a certain individual. From the scenarios, we learned that environments might vary a lot; therefore, we need to understand the environment where the situation happens and component-specific environments (eg, individual, services, stakeholders, and IT systems) to capture all privacy aspects. With the environment, we do not only mean location and other position-based information, but especially important is to capture the type of environment. We have to perceive the properties of environment such as privacy, security, trust-related information, and information-processing rules and responsibilities. Regulations may differ a lot between environments and different businesses are affected by their specific legislation. Capturing environment is crucial because technological advancements such as cloud computing and big data create new types of privacy risks. For example, if a service is offered in the European Union but the data are stored or processed in an information system located in the United States, there are differences in legislations concerning privacy, security, or secondary use of data. People should be able to control where and why their data are processed.

An individual component describes the actual subject and/or object of health and wellness activities in ubiquitous health. It is linked differently to situations; an individual can create them, participate in them, and/or is an object. Properties needed from the individual are the role he/she has in the situation, location, and environment and what relation he/she has with the activity. Also, an individual’s rights for controlling information processing (eg, content, disclosure and access to information), privacy policies, sensitivity and confidentiality requirements and what is his/her relation (eg, owner, controller, or subject) to information should be acknowledged. All these things affect how and on what basis systems can process information.

A service component describes regulated health care services and/or other services that can be offered by IT systems and/or stakeholders. An IT system component refers to all computational entities, which can include health information systems, personal health systems, ubiquitous systems, devices, sensors, etc. IT systems should be open about their processes and publish their privacy and security policies including how an individual’s privacy is protected, relevancy of processing and actual data protection specifications, and detailed information-processing principles. This would improve transparency of information processing and increase trustworthiness. If an IT system does not publish necessary information, this has to be captured in the context information. Because information processing can happen anywhere, it is vital to capture its context because there are several characteristics affecting privacy that may differ between IT systems; for example, type, location, or regulative background. For example, there are big differences in regulations among information systems, regulated medical devices (eg, have to be certified), and wellness devices. Stakeholder is the social component describing organizations and possible human participants. They can be actors or interested parties in a situation. They offer, participate, or are interested in services offered to individuals.

**Figure 3:**
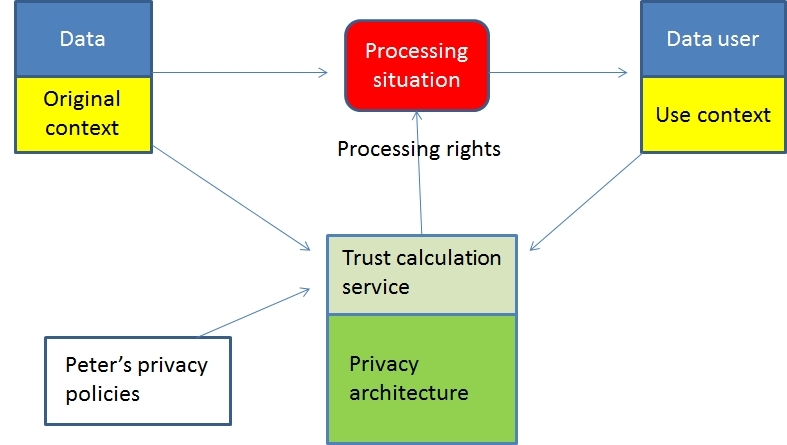
The use of privacy-related context information in ubiquitous health.

Our components can be used to increase trustworthiness of information processing because privacy policies can be adaptable and based on constraints, limitations, rules, rights, and responsibilities set with situational information. Components can also be used to analyze that the information processing follows the preferences set by an individual’s privacy policies and the requirements from the original context of the information. For example, in our user story Peter may disclose medical and lifestyle data to a service provider to receive a selected service. Peter has set privacy policies using privacy properties. Before disclosure, Peter (his privacy architecture) needs the context information from the service provider to calculate if processing is according to the requirements set by Peter’s own context-aware privacy policies and the original context of the information. Privacy architecture can then confirm that the use context is valid according to Peter’s personal preferences and allow access to the information (Figure 3).

Our hypothesis was that privacy-related context information could be used to formulate context-aware privacy policies hence enabling trusted processing of personal health and wellness information. In this study, we analyzed contents of a privacy attribute context and presented components and their properties that can be used as part of privacy policies by setting situational constraints and limitations. These characteristics are also needed to capture information-processing contexts from the privacy perspective. All components or properties are not necessarily needed in all situations. In addition, if some systems refuse to cooperate in publishing context information, this has to be captured and acknowledged.

## Discussion

In this research, we present an approach using privacy-related context information for privacy protection in ubiquitous health. Privacy is a business-enabler because individuals will not use these services if they cannot manage their privacy and trust. People need simple tools to manage their privacy and we have started this by defining the components situation, environment, individual, service, stakeholder, IT system, and their properties. These components describe the crucial privacy-related context information needed to improve trustworthiness of ubiquitous health. We present new knowledge by defining context, which is one of the main privacy attributes used in privacy policy definition. The results of this study can be used as a basis to create more formal models defining privacy-related context information in a computer-understandable format. Our results are in line with the preferred privacy level model by Lederer et al [11] but we have taken it a step further and divided context into original and use context and defined more detailed and concrete properties that could be valued and measured and used by privacy architecture for trust calculation.

Ubiquitous health is still an emerging field combining highly regulated health care with personal health and wellness services and systems. In health care, legislation and regulations define what privacy is and what kind of rights individuals have; that is, privacy is a state-defined property. Considering services and systems outside the regulated health care privacy is a personal property of an individual; that is, free will. The individual has the right to choose the use of his/her information and define policies as to how, where, and to what extent the information can be processed. In ubiquitous health, a privacy model is a combination of these two models and can be controlled with policies. Policies can be personal preferences or defined by regulations. Using the scenarios, we could identify situations outside regulated health care to recognize requirements and characteristics of ubiquitous health. With organization-centric health care processes or workflows, we cannot really model ubiquitous health as a whole because there are many services and systems without predefined and regulated processes or workflows.

In ubiquitous health, service provision is based on customer relationships and trading on benefits of services against reducing personal privacy. Individuals should be able to verify the trustworthiness of service providers and decide if they are prepared to disclose personal information and reduce privacy. Because services are often offered as distributed, personalized and even autonomous, the privacy architecture should offer automatic privacy services and adapt dynamically to the situation. Scenarios and user stories showed that ubiquitous health is multidimensional with limitations of predefining situations. The amount of information needed and created in these situations can be huge, and the content and its sensitivity vary depending on the activity performed. Ubiquitous health is an open, dynamic, and collaborative environment and privacy needs to be based on trust and its properties [19,21,24].

In health care, privacy is mainly protected with access control and consent management. Access control is merely one tool to protect privacy. Managing privacy in ubiquitous health is a much broader issue than just controlling health care professionals’ access to data. Access control with predefined rights, roles, and consents cannot really function because there is no central control or necessarily predefined processes, situations, or actors. To ensure privacy in ubiquitous computing, access control should be dynamic because of multiple changing entities. Context information enables dynamic management of rights [54]. Consent is an example of a personal policy but in ubiquitous health, policies are needed to cover several different situations that are more complex than those that consents are designed for. Policies have to be dynamic and context-aware. Corradi et al [55] present a dynamic and flexible security middleware that uses context as a basic concept in security policy specification and permissions are linked to the contexts instead of user identities or roles. Most research on privacy of context-aware computing focuses on capturing user’s context or certain actors and using that information to adapt to privacy preferences [23,47,54].

In this study, we followed the approach of Behrooz and Devlic [46] to separate situations and privacy rules. We identified the necessary information to capture privacy aspects in information-processing situations. Then, privacy architecture can capture the situation and the conditions where data are created; that is, the original context and combine that with individual's policies and control future use contexts such as how, where, and by whom the information can be used. Our approach needs information from participating systems and currently its availability depends on the goodwill of participants. Additional to this information, privacy architecture can use external sources for estimating trustworthiness of systems (eg, recommendations from others, history, trust values, and trust calculations).

In the European Union, organizations are required to inform individuals about use of their data and publish privacy policies that should be comprehensive with high-level descriptions of their privacy practices [43]; however, these are not enough to safeguard individuals’ rights. These privacy policies do not generally consider how data are actually processed after collection. So, one of the main challenges in privacy protection is how to enforce all relevant parties to explicate their detailed privacy policies [43]. Current legislation is not fully prepared to handle privacy threats of ubiquitous computing and does not obligate organizations to disclose their detailed privacy policies or information-processing principles. In the future, legislation needs to include the needs of privacy, citizens’ rights, and ubiquitous computing. Citizens have to be able to control processing and secondary use of their personal information. Future privacy principles and norms need to progress from high-level principles to detailed regulations concerning the processing and use of information. This would bring openness and transparency to information processing and new kinds of responsibilities for organizations and informed rights for citizens. In addition, authorities or certificate organizations should be able to audit providers and offer recommendations about their trustworthiness.

The components defined in this research may have some limitations and may not be conclusive; however, based on the scenario analyses these are needed. In addition, some properties are hard to define explicitly or in measurable format. They have to be analyzed in more detail and formal models are needed to implement them in computational format. Also, we need more detailed analysis of what organizations should publish about their processes and privacy and security policies and principles. To create context-aware privacy services and policies in practice, we need to develop ontologies that explicate components, properties, and requirements that we have presented in this research. Ontologies are formal representations and should cover different activities, services, IT systems, stakeholders, information content, and especially relevant regulative environments. With ontologies, we can create computational rules that can be used to enforce regulations and personal policies into ubiquitous applications.

Because it is practically impossible for individuals to evaluate the trustworthiness of a system, and to understand detailed privacy and security requirements and set personal policies, we developed trust-based privacy management architecture for ubiquitous health [8]. This architecture model describes what privacy and security services are needed to enable trusted information processing in ubiquitous health. The architecture will apply privacy-related context information to create privacy and security policies that will ensure that information processing will not happen against the wishes of the individual and the original context of the data. The architecture contains decision support and policy services for individuals to help them define personal policies. This research adds to the architecture model by defining the required privacy-related context information components and their properties that are needed to create implementable tools and means for individuals to manage personal information privacy.

## Abbreviations

IT: information technology
PHS: personal health system
